# Strong immunogenicity of heterologous prime-boost immunizations with the experimental vaccine GRAd-COV2 and BNT162b2 or ChAdOx1-nCOV19

**DOI:** 10.1038/s41541-021-00394-5

**Published:** 2021-11-04

**Authors:** Chiara Agrati, Stefania Capone, Concetta Castilletti, Eleonora Cimini, Giulia Matusali, Silvia Meschi, Eleonora Tartaglia, Roberto Camerini, Simone Lanini, Stefano Milleri, Stefano Colloca, Alessandra Vitelli, Antonella Folgori

**Affiliations:** 1 Istituto Nazionale per Le Malattie Infettive Lazzaro Spallanzani IRCCS, Rome, Italy; 2 ReiThera Srl, Rome, Italy; 3 Centro Ricerche Cliniche di Verona srl, Verona, Italy

**Keywords:** Vaccines, Public health

## Abstract

Here we report on the humoral and cellular immune response in eight volunteers who autonomously chose to adhere to the Italian national COVID-19 vaccination campaign more than 3 months after receiving a single-administration GRAd-COV2 vaccine candidate in the context of the phase-1 clinical trial. We observed a clear boost of both binding/neutralizing antibodies as well as T-cell responses upon receipt of the heterologous BNT162b2 or ChAdOx1-nCOV19 vaccines. These results, despite the limitation of the small sample size, support the concept that a single dose of an adenoviral vaccine may represent an ideal tool to effectively prime a balanced immune response, which can be boosted to high levels by a single dose of a different vaccine platform.

## Introduction

During the first year of the COVID-19 pandemic, several clinical trials provided evidence of the efficacy of a number of candidate vaccines against SARS-CoV-2^1^. However, with new COVID-19 waves and global vaccine shortages, the benefits of mixing two vaccines into one heterologous vaccination regimen are apparent and point toward a possibility of implementing this regimen to reach broad vaccine coverage^2^. A heterologous vaccination regimen may also be needed to maintain protective immunity against SARS-CoV-2 over time. Finally, the use of a different vaccine has been suggested as a way to overcome hesitancy in persons who received a first dose of an adenoviral-vectored vaccine for which safety concerns were raised. In fact, several European countries, such as Spain and Germany, have implemented a booster dose with mRNA vaccines to people under the age of 60 that received primary vaccination with ChAdOx1-nCOV19.

There is experimental evidence suggesting that heterologous vaccination improves the immune responses^3^ and indeed this strategy has been proposed in vaccination against other viruses^4^ and cancer^5^. Preclinical data in mice confirm the immunological benefit of combining COVID-19 vaccines from different platforms^6,7^ and several clinical trials are presently underway to test the safety and immunogenicity of this strategy for SARS-CoV2 infection (NCT04907331, NCT04962906, NCT04760730, NCT04684446, and NCT0477631).

## Results

### Humoral and cell-mediated immune response after heterologous vaccination

We recently concluded a Phase-1 study to assess the safety and immunogenicity of a single-dose regimen of a gorilla adenovirus-vectored vaccine (GRAd-COV2) in younger (aged 18–55) and older (65–85) volunteers^8^. The results showed good safety of the GRAd-COV2 vaccine and induction of both humoral (binding as well as neutralizing antibodies) and Th1-skewed cell-mediated immune response. At the final 24-week monitoring visit, eight enrolled volunteers reported that, outside the study procedures and in the context of the Italian National Vaccination Campaign (Table 1), they received either a full course (*n* = 3) or only the first dose of BNT162b2-mRNA (*n* = 3), or one dose of ChAdOx1 (*n* = 2). These vaccinations occurred in a time frame between 14 and 24 weeks after the GRAd-COV2 study vaccination, and therefore the side effects were not collected. These subjects provided blood samples to perform protocol-defined laboratory test on anti-SARS-CoV-2 humoral and cell-mediated immune response.

**Table 1:** Volunteers characteristics and vaccination history.

	Volunteer id	Gender/age range	study arm	GRAd-COV2 dose (vp^a^)	Pfizer/AZ dose(s) study week
Pfizer x 2	A	M/26–30 y	2B	1 × 10^11^ vp	Pfizer x 2, week 16 &19
Pfizer x 2	B	M/21–25 y	3B	2 × 10^11^ vp	Pfizer x 2, week 19 & 22
Pfizer x 2	C	M/76–80 y	5B	1 × 10^11^ vp	Pfizer x 2, week 14 &−17
Pfizer x 1	D	M/71–75 y	4B	5 × 10^10^ vp	Pfizer x 1, week 22
Pfizer x 1	E	M/71–75 y	6B	2 × 10^11^ vp	Pfizer x 1, week 17†
Pfizer x 1	F	M/21–25 y	2B	1 × 10^11^ vp	Pfizer x 1, week 24
ChAdOx1 ×1	G	M/76–80 y	4B	5 × 10^10^ vp	AZ x 1, week 21
ChAdOx1 ×1	H	M/71–75 y	5B	1 × 10^11^ vp	AZ x 1, week 21

^a^vp = viral particles; † final study visit (w24) anticipated at week 20

**Fig. 1:**
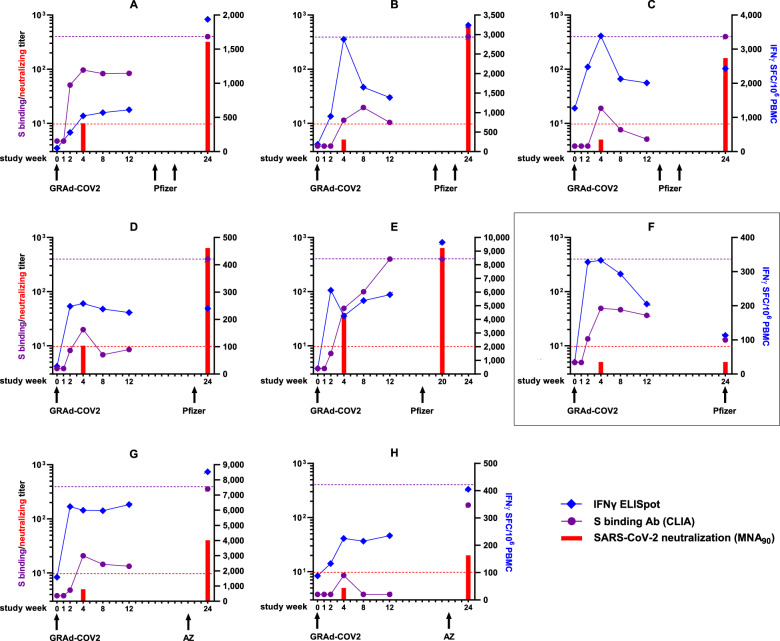
Immunogenicity profile of GRAd-COV2-vaccinated volunteers receiving approved COVID-19 vaccines. Each graph corresponds to an individual volunteer, as indicated by the code in the graph title. Subjects who received 2 doses of Pfizer (Panels A-C), 1 dose of Pfizer (Panels D-F) or 1 dose of ChAdOx1 (Panels G-H) are shown. Arrows at the graph bottom indicate study week when each GRAd-COV2 or Pfizer (BNT162b2)/AZ (ChAdOx1-nCoV19) vaccination was received. Numbers on the *x* axis indicate timing in weeks from GRAd-COV2 vaccination when a study visit occurred. The purple round symbols indicate Spike-binding IgG levels, as measured by CLIA assay and expressed in arbitrary units (AU)/ml. The red bars at w4 and 24 show SARS-CoV-2-neutralizing antibody titers, expressed as MNA_90_. Both serological endpoints are plotted against the left *y* axis. Purple dotted line set at 400 AU/ml indicates the upper limit of quantification for CLIA. Dotted red line indicates positivity threshold for the MNA assay, i.e., a neutralization titer of 1:10 or higher is deemed positive. The blue diamond symbols show Spike-specific T-cell response as measured by IFNγ ELISpot, expressed as IFNγ spot forming cells (SFC)/million PBMC, and plotted against the right *y* axis. The box highlights the subject F, receiving the booster dose only three days before the week-24 visit.

In response to the heterologous vaccines, the levels of anti-Spike-binding antibodies measured at week 24 were greatly amplified compared with week 12, and exceeded the peak level recorded in each individual around week 4–8 post GRAd-COV2 vaccination (Fig. 1), with no major differences in subjects receiving one or two doses of the heterologous vaccine. The only exception is volunteer F, who received the first BNT162b2 only three days before the week-24 visit, representing an ideal internal “no-boost” control. Indeed, Spike Ab response slightly contracted in volunteer F from the last week-12 visit. Similarly, neutralizing titers at week 24 (MNA_90_) were potently boosted in all volunteers, except F, and clearly exceeded those measured at study week 4. T-cell responses were also assessed throughout the study visits by IFNγ ELISpot on frozen PBMC. Interestingly, the strong T-cell response induced by GRAd-COV2 was further boosted with respect to both the peak and/or the week-12 levels in two volunteers receiving two mRNA doses (A and B), one receiving one mRNA dose (E), and in two volunteers receiving one ChAdOx1 dose (G and E). In the remaining subjects receiving one (D) or two mRNA doses (C), the T-cell responses at week 24 were maintained at the same level as three months before, but did not further contract as observed in F.

## Discussion

This report has obvious limitations in the number of subjects and in the lack of safety data of the heterologous administration. Nevertheless, our observations suggest that the immune responses induced by the single-dose GRAd-COV2 vaccination can be effectively boosted also by a single heterologous vaccination with two approved vaccines, amplifying both antibody and T-cell response.

These data are confirmatory of recently disclosed results from a study of safety and immunogenicity of homologous and heterologous immunization with ChAdOx1-nCoV19 and BNT162b2^9^, as well as initial immunogenicity data from a randomized noninferiority trial comparing heterologous and homologous regimens of the same two vaccines (Com-COV study^10^).

Overall, these evidences support the concept that a single dose of an adenoviral vaccine, which is cheap and easy to deploy in a pandemic setting, represents a good tool to effectively prime the immune system, which can be boosted afterward by a single dose of a different vaccine based on a diverse (mRNA or protein in adjuvant) or similar (human or simian Ad) platform, ultimately reaching high levels of immune responses. As preclinical and clinical evidence suggest^6,9^, keeping adenoviral-vectored vaccines as part of heterologous vaccination regimens may be the key for optimal induction and persistence of T-cell responses, a feature that may confer cross-protection in the context of already-circulating and newly emerging SARS-CoV-2 variants^11^. Further prospective and controlled investigations are mandatory to confirm these data and to evaluate the impact of age and the optimal timing between prime and boost, as well as the order of administration of the heterologous vaccines.

## Methods

### Study design

RT-CoV-2 is a phase-1, dose-escalation, open-label clinical trial in two cohorts of healthy younger (18–55) or older (65–85) adults. Each age cohort consisted of 3 arms of 15 volunteers each, to assess the safety and immunogenicity of a single GRAd-COV2 intramuscular administration at three different dose levels: 5 × 10^10^ viral particles (vp) (arms 1—younger and 4—older), 1 × 10^11^ vp (arms 2—younger and 5—older), and 2 × 10^11^ vp (arms 3—younger and 6—older). All participants provided written informed consent before enrolment. The trial was conducted at the National Institute for Infectious Diseases Lazzaro Spallanzani (INMI) in Rome and at Centro Ricerche Cliniche in Verona (CRC-Verona), according to the Declaration of Helsinki, and approved by the Italian Regulatory Drug Agency (AIFA) and the Italian National Ethical Committee for COVID-19 clinical studies (ClinicalTrials.gov NCT04528641; EudraCT 2020-002835-31). Further information on study design and interim analysis safety and immunogenicity data can be found in^8^. A phase-2 trial of GRAd-COV2 vaccine is currently ongoing (NCT04791423).

### SARS-CoV-2 anti-Spike IgG high-throughput chemiluminescence immunoassay

Serum was collected at planned study visits the day of vaccination (week 0) and 1, 2, 4, 8, 12 and 24 weeks later. Spike-binding IgG was measured using a chemiluminescence immunoassay (CLIA), namely DiaSorin LIAISON^®^ SARS-CoV-2 S1/S2 IgG test on LIAISON^®^ XL analyzers (DiaSorin, Italy), following the manufacturer’s instructions. IgG concentrations are expressed as arbitrary units, AU/mL. According to the datasheet, results >15 are clearly positive, between 12 and 15 are equivocal, and <12 are negative or may indicate low level of IgG antibodies to the pathogen.

### SARS-CoV-2 microneutralization assay (MNA)

Neutralizing antibodies to SARS-CoV-2 were assessed by a microneutralization assay with live SARS-CoV-2 virus (strain 2019-nCoV/Italy-INMI1; GISAID accession ID: EPI_ISL_412974). The assay is described in detail in^8^ and is based on inhibition of Vero E6 cell infection by serum-dilution curves, with cytopathic-effect (CPE) determination at 48 h post infection. The highest serum dilution inhibiting at least 90% of the CPE was indicated as the neutralization titer and expressed as the reciprocal of serum dilution (MNA_90_). Briefly, heat-inactivated and titrated sera (duplicate twofold serial dilutions, starting dilution 1:10) were mixed with equal volumes of 100 TCID_50_ SARS-CoV-2 and incubated at 37 °C 5% CO_2_ for 30 min. Subsequently, 96-well tissue culture plates with subconfluent Vero E6 cell monolayers were infected with 100 μl/well of virus–serum mixtures and incubated at 37 °C and 5% CO2. To standardize interassay procedures, positive-control samples showing high (1:160) and low (1:40) neutralizing activity were included in each MNA session. After 48 h, microplates were observed by light microscope for the presence of CPE and then stained with Crystal Violet solution containing 2% formaldehyde. Cell viability was measured by a photometer at 595 nm (Synergy™ HTX Multi-Mode Microplate Reader, Biotek). The highest serum dilution inhibiting at least 90% of the CPE was indicated as the neutralization titer and expressed as the reciprocal of serum dilution (MNA_90_).

### IFNγ ELISpot assay on cryopreserved PBMC

The magnitude and kinetics of vaccine-induced Spike-specific T cells, as a function of gamma-interferon (IFNγ) production in response to antigen restimulation, was assessed by standard IFNγ ELISpot. Peripheral blood mononuclear cells (PBMC) were isolated by standard Histopaque (Sigma Aldrich) gradient technique the day of vaccination (week 0) and 2, 4, 8, 12, and 24 weeks later. Cryopreserved PBMC were thawed and rested overnight at 37 °C in R10 medium [RPMI 1640 (Sigma Aldrich) supplemented with 10% heat-inactivated highly defined fetal bovine serum (FBS-HyClone), 2 mmol/L L-glutamine, 10 mmol/L HEPES buffer (N-2-hydroxyethylpiperazine-N-2-ethane sulfonic acid, Sigma Aldrich), 100 U/ml penicillin, and 100 µg/mL streptomycin (Gibco)]. Rested PBMC were plated at 2 × 10^5^ cells/well in ELISpot plates (Human IFN-γ ELISpot plus kit; Mabtech) and stimulated for 18–20 h with 15-mer peptides overlapping by 11 amino acids covering the full-length Spike protein (synthetized by Elabscience Biotech Inc, distributed by TEMA RICERCA), arranged in 2 pools: S1 and S2 (3 μg/ml final concentration of each peptide). At the end of incubation, ELISpot assay was developed according to the manufacturer’s instructions. Spontaneous cytokine production (background) was assessed by incubating PBMC with DMSO, the peptide diluent (Sigma). The results are expressed as spot-forming cells (SFC)/10^6^ PBMCs in stimulating cultures, by summing responses to S1 and S2 after subtracting background.

### Reporting summary

Further information on research design is available in the Nature Research Reporting Summary linked to this article.

## Supplementary information

Reporting Summary

## Data Availability

All data generated or analyzed during this study are included in this published article.
