# Supplementary material for: Strong immunogenicity of heterologous prime-boost immunizations with the experimental vaccine GRAd-COV2 and BNT162b2 or ChAdOx1-nCOV19
Source: NPJ Vaccines. 2021 Nov 4;6:131. doi: 10.1038/s41541-021-00394-5 (PMC8569156; doi:10.1038/s41541-021-00394-5)
Supplement: Supplementary file 1 — Reporting Summary [file 41541_2021_394_MOESM1_ESM.pdf]

nature portfolio

Corresponding author(s): Dr CHIARA AGRATI

Last updated by author(s): 02/09/2021

Reporting Summary

Nature Portfolio wishes to improve the reproducibility of the work that we publish. This form provides structure for consistency and transparency in reporting. For further information on Nature Portfolio policies, see our Editorial Policies and the Editorial Policy Checklist.

Statistics

For all statistical analyses, confirm that the following items are present in the figure legend, table legend, main text, or Methods section.

n/a	Confirmed	
☒	☐	The exact sample size (*n*) for each experimental group/condition, given as a discrete number and unit of measurement
☒	☐	A statement on whether measurements were taken from distinct samples or whether the same sample was measured repeatedly
☒	☐	The statistical test(s) used AND whether they are one- or two-sided<br>*Only common tests should be described solely by name; describe more complex techniques in the Methods section.*
☒	☐	A description of all covariates tested
☒	☐	A description of any assumptions or corrections, such as tests of normality and adjustment for multiple comparisons
☒	☐	A full description of the statistical parameters including central tendency (e.g. means) or other basic estimates (e.g. regression coefficient) AND variation (e.g. standard deviation) or associated estimates of uncertainty (e.g. confidence intervals)
☒	☐	For null hypothesis testing, the test statistic (e.g. F, t, r) with confidence intervals, effect sizes, degrees of freedom and P value noted<br>*Give P values as exact values whenever suitable.*
☒	☐	For Bayesian analysis, information on the choice of priors and Markov chain Monte Carlo settings
☒	☐	For hierarchical and complex designs, identification of the appropriate level for tests and full reporting of outcomes
☒	☐	Estimates of effect sizes (e.g. Cohen's *d*, Pearson's *r*), indicating how they were calculated

Our web collection on statistics for biologists contains articles on many of the points above.

Software and code

Policy information about availability of computer code

Data collection	*Provide a description of all commercial, open source and custom code used to collect the data in this study, specifying the version used OR state that no software was used.*
Data analysis	*Provide a description of all commercial, open source and custom code used to analyse the data in this study, specifying the version used OR state that no software was used.*

For manuscripts utilizing custom algorithms or software that are central to the research but not yet described in published literature, software must be made available to editors and reviewers. We strongly encourage code deposition in a community repository (e.g. GitHub). See the Nature Portfolio guidelines for submitting code & software for further information.

Data

Policy information about availability of data

All manuscripts must include a data availability statement. This statement should provide the following information, where applicable:

- Accession codes, unique identifiers, or web links for publicly available datasets
- A description of any restrictions on data availability
- For clinical datasets or third party data, please ensure that the statement adheres to our policy

All data generated or analyzed during this study are included in this published article

Field-specific reporting

Please select the one below that is the best fit for your research. If you are not sure, read the appropriate sections before making your selection.

☒ Life sciences ☐ Behavioural & social sciences ☐ Ecological, evolutionary & environmental sciences

For a reference copy of the document with all sections, see nature.com/documents/nr-reporting-summary-flat.pdf

Life sciences study design

All studies must disclose on these points even when the disclosure is negative.

Sample size	The paper describes the immunogenicity of a heterologous boost vaccination schedule on eight subjects
Data exclusions	no data were excluded
Replication	all data shown in the manuscript derive from a single testing session
Randomization	not relevant-the original clinical trial is a phase 1 dose escalation where no randomization was adopted
Blinding	not relevant - the original clinical trial is an open lable phase 1 trial

Reporting for specific materials, systems and methods

We require information from authors about some types of materials, experimental systems and methods used in many studies. Here, indicate whether each material, system or method listed is relevant to your study. If you are not sure if a list item applies to your research, read the appropriate section before selecting a response.

Materials & experimental systems

n/a	Involved in the study
☒	☐ Antibodies
☒	☐ Eukaryotic cell lines
☒	☐ Palaeontology and archaeology
☒	☐ Animals and other organisms
☐	☒ Human research participants
☒	☐ Clinical data
☒	☐ Dual use research of concern

Methods

n/a	Involved in the study
☒	☐ ChIP-seq
☒	☐ Flow cytometry
☒	☐ MRI-based neuroimaging

Human research participants

Policy information about studies involving human research participants

Population characteristics	Eight volunteers enrolled in a Phase 1 study of a single-dose regimen of a gorilla adenovirus vectored vaccine (GRAd-COV2). These subjects, outside the study procedures and in the context of the Italian National Vaccination Campaign (Table 1), received either a full course (n=3) or only the first dose of BNT162b2-mRNA (n=3), or one dose of ChAdOx1 (n=2). The subjects were all male by chance (even if the original trial enrolled volunteers of both sexes), and with ages ranging between 21 and 80.
Recruitment	It was an autonomous decision of the eight subjects to enter National COVID-19 campaign before the end of the clinical trial follow up
Ethics oversight	Italian National Ethical Committee for COVID-19 clinical studies

Note that full information on the approval of the study protocol must also be provided in the manuscript.
